# Repetitive Long-Term Hyperbaric Oxygen Treatment (HBOT) Administered after Experimental Traumatic Brain Injury in Rats Induces Significant Remyelination and a Recovery of Sensorimotor Function

**DOI:** 10.1371/journal.pone.0097750

**Published:** 2014-05-21

**Authors:** Klaus Kraitsy, Muammer Uecal, Stefan Grossauer, Lukas Bruckmann, Florentina Pfleger, Stefan Ropele, Franz Fazekas, Gerda Gruenbacher, Silke Patz, Markus Absenger, Christian Porubsky, Freyja Smolle-Juettner, Irem Tezer, Marek Molcanyi, Ulrike Fasching, Ute Schaefer

**Affiliations:** 1 Research Unit for Experimental Neurotraumatology, Medical University of Graz, Graz, Austria; 2 Department of Neurosurgery, Medical University of Graz, Graz, Austria; 3 Clinical Division of General Neurology, Medical University of Graz, Graz, Austria; 4 Core Facility Microscopy, Centre for Medical Research, Medical University of Graz, Graz, Austria; 5 Division of Thoracic and Hyperbaric Surgery, Department of Surgery, Medical University of Graz, Graz, Austria; 6 Department of Neurosurgery, University of Cologne, Cologne, Germany; 7 Institute of Neurophysiology, University of Cologne, Cologne, Germany; Georgia Regents University, United States of America

## Abstract

Cells in the central nervous system rely almost exclusively on aerobic metabolism. Oxygen deprivation, such as injury-associated ischemia, results in detrimental apoptotic and necrotic cell loss. There is evidence that repetitive hyperbaric oxygen therapy (HBOT) improves outcomes in traumatic brain-injured patients. However, there are no experimental studies investigating the mechanism of repetitive long-term HBOT treatment-associated protective effects. We have therefore analysed the effect of long-term repetitive HBOT treatment on brain trauma-associated cerebral modulations using the lateral fluid percussion model for rats. Trauma-associated neurological impairment regressed significantly in the group of HBO-treated animals within three weeks post trauma. Evaluation of somatosensory-evoked potentials indicated a possible remyelination of neurons in the injured hemisphere following HBOT. This presumption was confirmed by a pronounced increase in myelin basic protein isoforms, PLP expression as well as an increase in myelin following three weeks of repetitive HBO treatment. Our results indicate that protective long-term HBOT effects following brain injury is mediated by a pronounced remyelination in the ipsilateral injured cortex as substantiated by the associated recovery of sensorimotor function.

## Introduction

The mammalian central nervous system relies almost exclusively on aerobic metabolism and demonstrates an extensive oxygen demand [1], [2]. The high metabolic rate of the brain has largely been associated with activity-dependent neuronal signalling processes such as synaptic and action potentials or excitatory neurotransmission [3]–[6]. Due to the high rate of neuronal-dependent oxidative metabolism and the lack of cerebral energy reserves, oxygen deprivation, such as injury-induced hypoxia or ischemia, results in the development of detrimental secondary brain damage, in particular apoptotic and necrotic neuronal cell death [7], [8].

In traumatic brain injury, cerebral tissue oxygenation has been shown to be associated with patient prognosis [9] as well as physiological outcome parameters [10]. Consequently, clinical efforts aim to keep brain tissue oxygenation above certain thresholds [11]. The means to achieve this include critical care interventions [11]–[13] as well as normobaric or hyperbaric oxygen treatments as complementary therapeutic approaches. Tissue oxygenation of the injured brain has been demonstrated to be most effective during hyperbaric oxygen application with supraphysiological levels exceeding 200 mm HG [14]. However, an explicit benefit of hyperbaric oxygen treatment for traumatic brain-injured patients has yet to be established, since standardized clinical studies reporting HBOT-associated protective effects on TBI mediated brain damage are scarce.

Many reports demonstrating an HBOT-associated reduction in mortalities or improvement of neurological outcomes post-TBI are based on case studies [15]–[18] or retrospective analyses [19]–[21]. In a prospective randomized study, Rockswold and colleagues demonstrated the robust but transient treatment effect of HBOT on cerebral blood flow, cerebral metabolic rate of oxygen and intracranial pressure in patients (n = 69) with sustained severe TBI [14]. O_2_ toxicity was not observed. These results were in line with those from an earlier study encompassing 37 non-randomized severely brain-injured patients [22]. HBOT-related complications, such as tension pneumocephalus, were described in case studies [23].

Patient heterogeneity as well as differences in study design and treatment modalities make the direct comparison of these results difficult. Although experimental studies are also based on varying animal models of brain injury and different HBO treatment modalities, the standardized set up of these studies allows for a more detailed exploration of the protective or detrimental effects of HBOT.

HBO treatment following experimental brain trauma was demonstrated to support recovery of aerobic metabolic function [24], an increase in overall cerebral glucose metabolism and cerebral PO_2_ [25], [26], an increase in vascular density and spatial learning [27], a significant reduction of apoptosis, higher cell counts as well as a denser axonal network [28], [29]. Recent studies also reported an HBOT-mediated stimulation of angiogenesis and neurogenesis following TBI [30], [31].

Thus far, experimental observations have primarily been based on short-term studies during the acute phase of injury. Generally, HBO treatment was started within 3 hours of brain injury and pursued for a maximum of five days with the number of HBOT treatments varying between 1 and 6 sessions [24]–[26], [28], [29], [32]–[38]. At the same time, however, neurological improvements following TBI in the clinical setting have often been reported following 20 to 120 HBOT sessions [17], [39]–[44].

We therefore hypothesized that repetitive long-term HBO treatment following traumatic brain injury would have more pronounced effects and might modulate different cerebral functions than short-term treatment during the acute phase of TBI only. To test this hypothesis, we analysed the effects of repetitive HBOT treatment applied over a 3-week period following moderate and severe lateral fluid percussion head injuries. The HBOT-mediated modulation of sensorimotor functions and of somatosensory-evoked potentials was evaluated every week. Due to a pronounced decrease in the central conduction time in the ipsilateral hemispheres following four weeks of HBOT treatments, the myelination of neurons in the injured hemispheres was investigated in more detail.

Our results indicate that long-term HBO treatment following brain injury mediates pronounced remyelination in the ipsilateral cortex, which in turn, is associated with a significant increase in motor function improvement.

## Results

### Long-term HBOT schedule and data analysis

**Figure 1:**
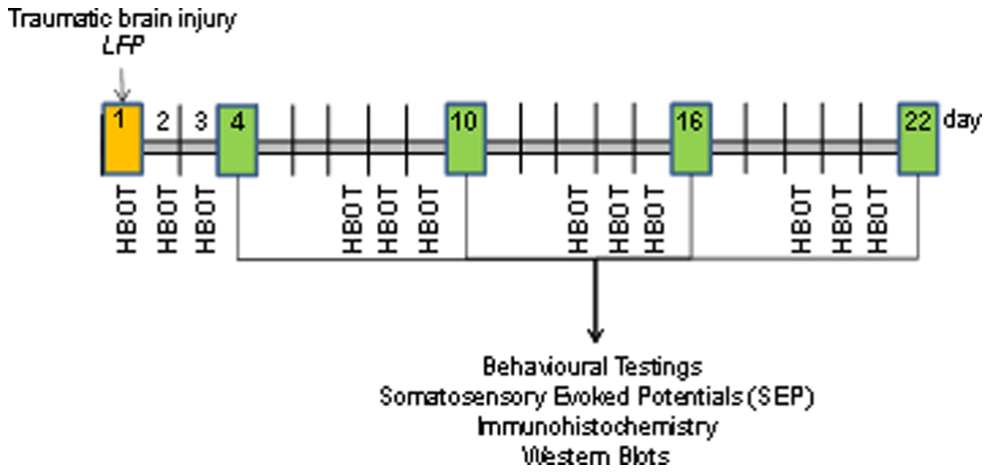
Schematic representation of the schedule of HBO treatments and associated analytical assays following induction of traumatic brain injury by fluid percussion.

HBO treatment (100% oxygen at 2.2 atm) was implemented 1 hour after induction of TBI. Each treatment lasted 1 hour. The procedure was repeated on days 2 and 3 after injury induction and at weeks 1, 2 and 3 for a further 3 days each (Fig. 1). Morphological changes, somatosensory-evoked potentials (SSEP), protein expression and behavioural tests were analysed following the three successive HBO treatments at days 4, 10, 16 and 22.

Correlation between trauma severity and outcome were assessed for different parameters. At a correlation coefficient of r>0.55 or r<−0.55, results were grouped by either moderate (<2.5 atm) or severe injuries (>2.5 atm). For correlation coefficients r<0.55 or r>−0.55 that indicated severity-independent results, the data from the different groups were combined. Trauma severity-associated changes were only observed for the analysis of neurological motor function by the Rotarod performance test and the composite neuroscore testing paradigm (for correlation analysis see Fig. S1, S2 and S4).

### Effect of long-term HBOT on TBI-induced morphological and structural damage

**Figure 2:**
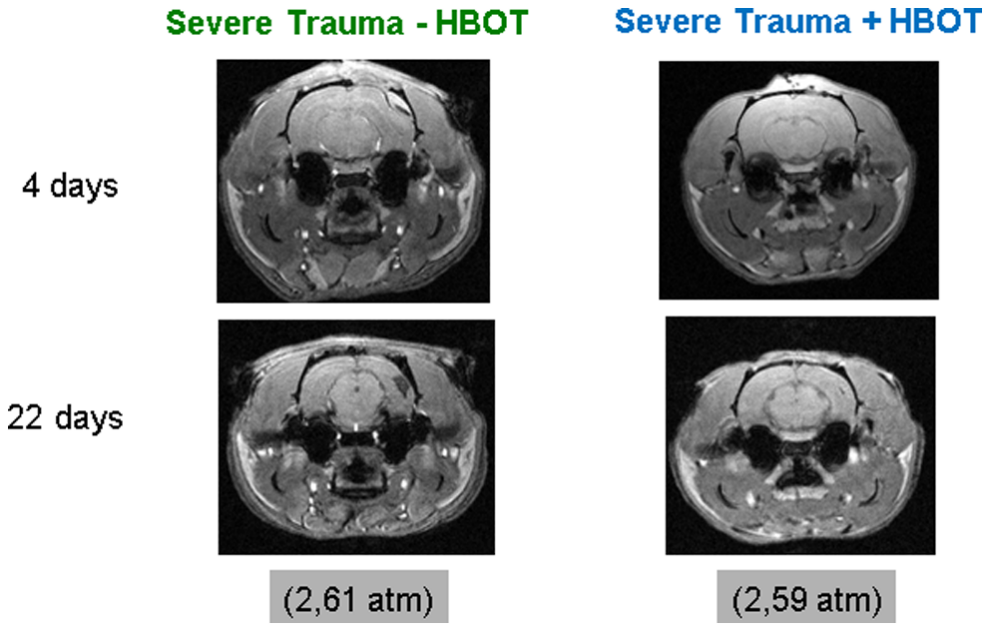
Representative coronal sections through a rat brain from the T1-weighted gradient echo sequence at days 4 and 22 following trauma and repetitive HBO treatment. The lesions observed in the ispilateral cortex of HBO-treated animals appear less pronounced. The in-plane resolution was 200×200 µm^2^ and the slice thickness was 500 µm.

Traumatic brain injury results in progressive long-term functional and structural brain damage due to elevated inflammation, suppressed regeneration and cell death. 22 days following lateral fluid percussion, ipsilateral structural damage such as cortical lesions or ventricle atrophy was detectable by magnetic resonance imaging. This is in accordance with our previous results documenting pronounced ipsilateral cerebral damage within 3 weeks following traumatic brain injury [53]. HBOT-associated reduction in these lesions was also observable at this time point. (Fig. 2). Due to the variability in the trauma-induced lesions, quantitative analysis of the structural damage was not possible. In Table S1 the number of animals with or without distinct lesions in the different groups is summarized.

### Effect of long-term HBOT on TBI-induced modulations in SSEP

Trauma-associated extensive ipsilateral cortical damage might be accompanied by the impaired integrity of the central neuronal pathways. In order to assess whether the HBOT-associated decrease in TBI induced lesions/structural damage was linked to an improvement of cerebral function, focal interruptions along the somatosensory pathway were quantified. Amplitude-latency analysis was used to assess somatosensory-evoked potentials (SSEP).

**Figure 3:**
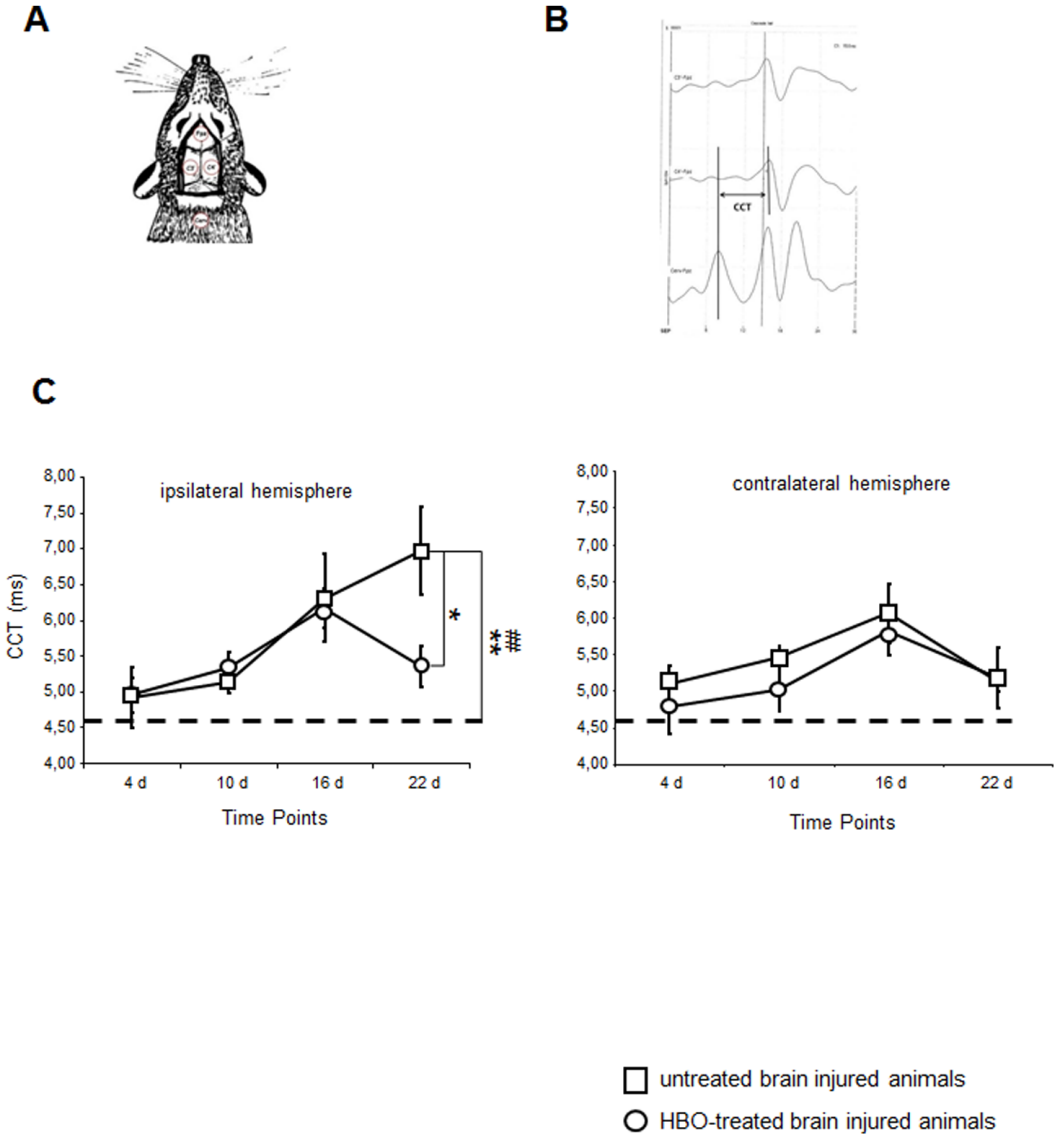
Time-dependent changes in the central conduction times following traumatic brain injury and HBO treatment established by somatosensory-evoked potentials. A. Schematic representation of the somatosensory-evoked potential recording sites adapted from the human 10–20 system. B. The central conduction time (CCT) is calculated by subtracting the peak latency of the major response from the neck from the latency of the primary cortical response C. Time-dependent modulation in the central conduction time recorded at the ipsi- and contralateral hemisphere in treated and untreated brain injured rats. Open square: HBO-treated brain injured rats; open circle: untreated brain injured rats; ---- baseline recordings; The HBOT effect was not observed at all time points (repeated measures mixed model ANOVA; *F(1,20) = 0.38, n.s.*); however, there was a significant effect at 3 weeks. *p<0.05 (repeated measures ANOVA, Bonferonni corrected); ^#^ *p*≤0.003 (t-test), HBO-treated versus untreated injured animals. ^##^ *p<0.001* (ANOVA), HBO-treated animals versus sham controls; n = 5 for untreated, n = 12 for HBO-treated (uneven group sizes arise from missing data points in some animals, which were excluded in repeated measures mixed model ANOVA). Error bars represent ± standard error means.

After collecting complete SSEP data, the stored recordings were evaluated for the presence of the main cervical potential in the channel Cerv-Fpz and the primary cortical potentials in the channels of C3'-Fpz and C4'-Fpz (Fig. 3A); the central conduction time (CCT) was then calculated in all cases of present potentials by subtracting the peak latency of the major response of the neck from that of the primary cortical response, as shown in Fig. 3B.

The mean CCT of sham animals as well as brain-injured animals before induction of trauma (baseline) was recorded at 4.55 ms. CCT values obtained from the ipsilateral side (C4'-Fpz) increased in treated and untreated animals within 4 days post-trauma (Fig. 3C). Two weeks post-trauma, the mean conduction time was delayed by 2.25 ms in treated and untreated animals as compared to sham animals. At three weeks post-trauma CCT values worsened significantly in brain-injured animals. The CCT of HBOT-treated animals decreased significantly (Fig. 3C). A similar time-dependent pattern of CCT modulations was observed in the contralateral hemisphere at the early time points of observation. However, a spontaneous recovery of the central conduction times was observed in the contralateral hemisphere that was comparable to the HBO treatment-induced changes in the ipsilateral hemisphere (Fig. 3C).

### Effect of long-term HBOT on TBI-induced modulations in cerebral myelination

**Figure 4:**
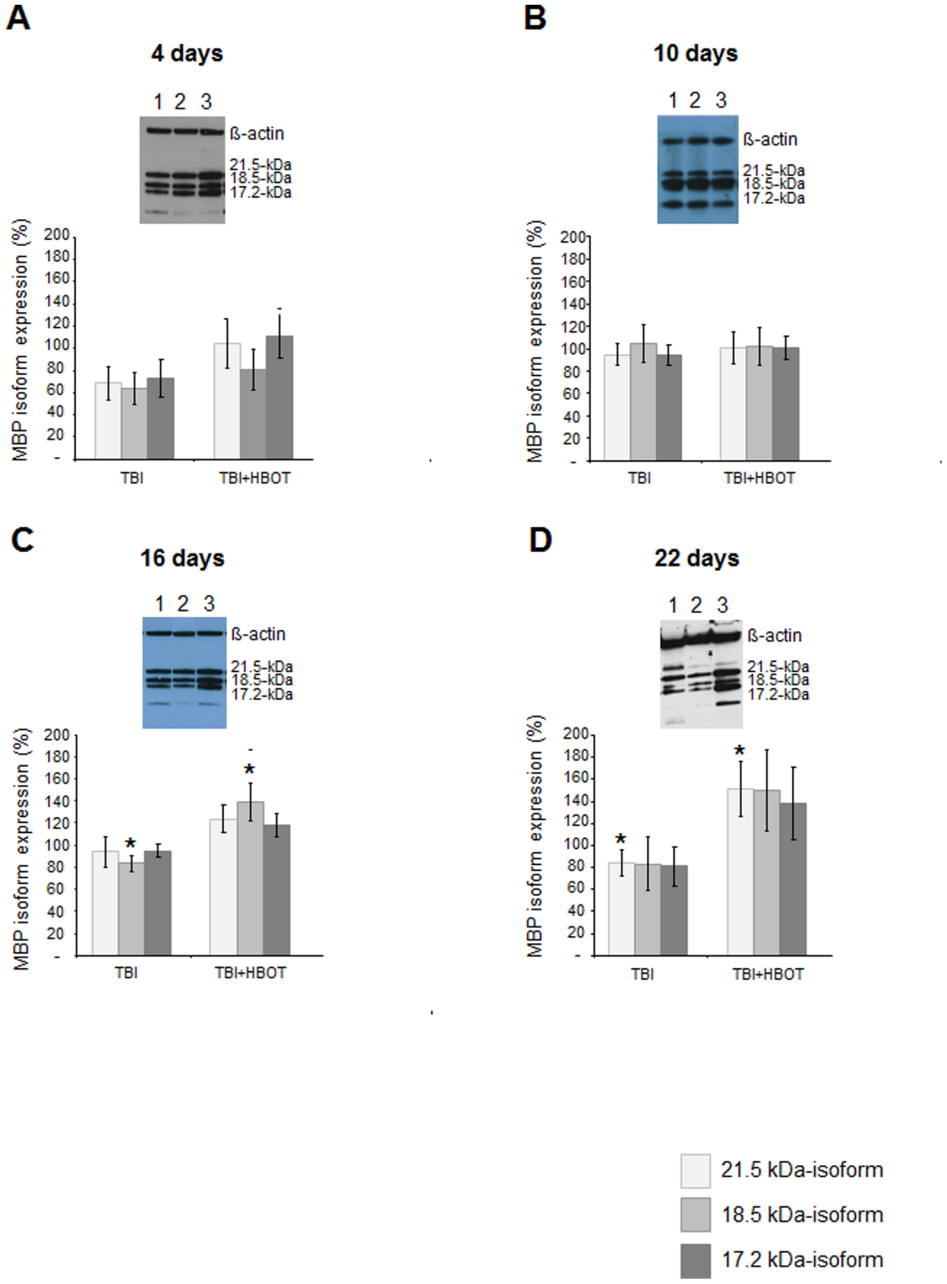
Western blot analysis of time-dependent changes in myelin basic protein isoform expression in the ipsilateral cortex following traumatic brain injury and HBO treatment. A-D Myelin basic protein isoform expression at days 4, 11, 16 and 22 as percentage of expression in sham controls and representative western blot gels. Light grey bars: expression of isoform 21.5-kDa, grey bar: expression of isoform 18.5-kDa; dark grey bar: expression of isoform 17.2-kDa isoform; insert:, 1: sham, 2: brain injured animals, 3: injured and HBO-treated animals; western blot analysis was repeated at least twice per animal sample; n≥4 for each group. *p<0.05, HBO treated vs untreated injured animals; ^#^ *p<0.05* as compared to sham controls. Error bars represent ± standard error means.

Myelin basic proteins (MBP) are essential constituents of the myelin sheath. There are several size isoforms of MBP formed by differential splicing of seven exons. In rats, MBP constitute the 21.5-, 17.2-, 18.5- and 14-kDa isoforms. We observed an injury-associated time-dependent biphasic reduction in the expression of MBP isoforms 21.5-, 17.2- as well as 18.5-kDa in the ipsilateral cortex at day 4 and day 22 post injury. The only statistically significant trauma-induced decrease of MBP expression as compared to sham controls was detected for isoform 18.5-kDa at day 4 (Fig. 4A-D; p<0.05). A distinct pattern of MBP isoform 14-kDa expression was not established. Statistically expression analysis of this isoform has thereby been omitted. An HBOT-mediated up-regulation of MBP isoform expression was observed at days 16 and 22 post-injury. Upregulation of isoform 18.5-kDa was statistically significant at day 16 (Figure 4C; p<0.01) as compared to untreated animals. However, upregulation of isoform 21.5-kDa was significant at day 22 (Figure 4D; p<0.05) as compared to sham controls as well as untreated animals indicating that expression of 21.5-kDa isoform in HBOT-treated animals to exceed isoform expression in sham controls.

**Figure 5:**
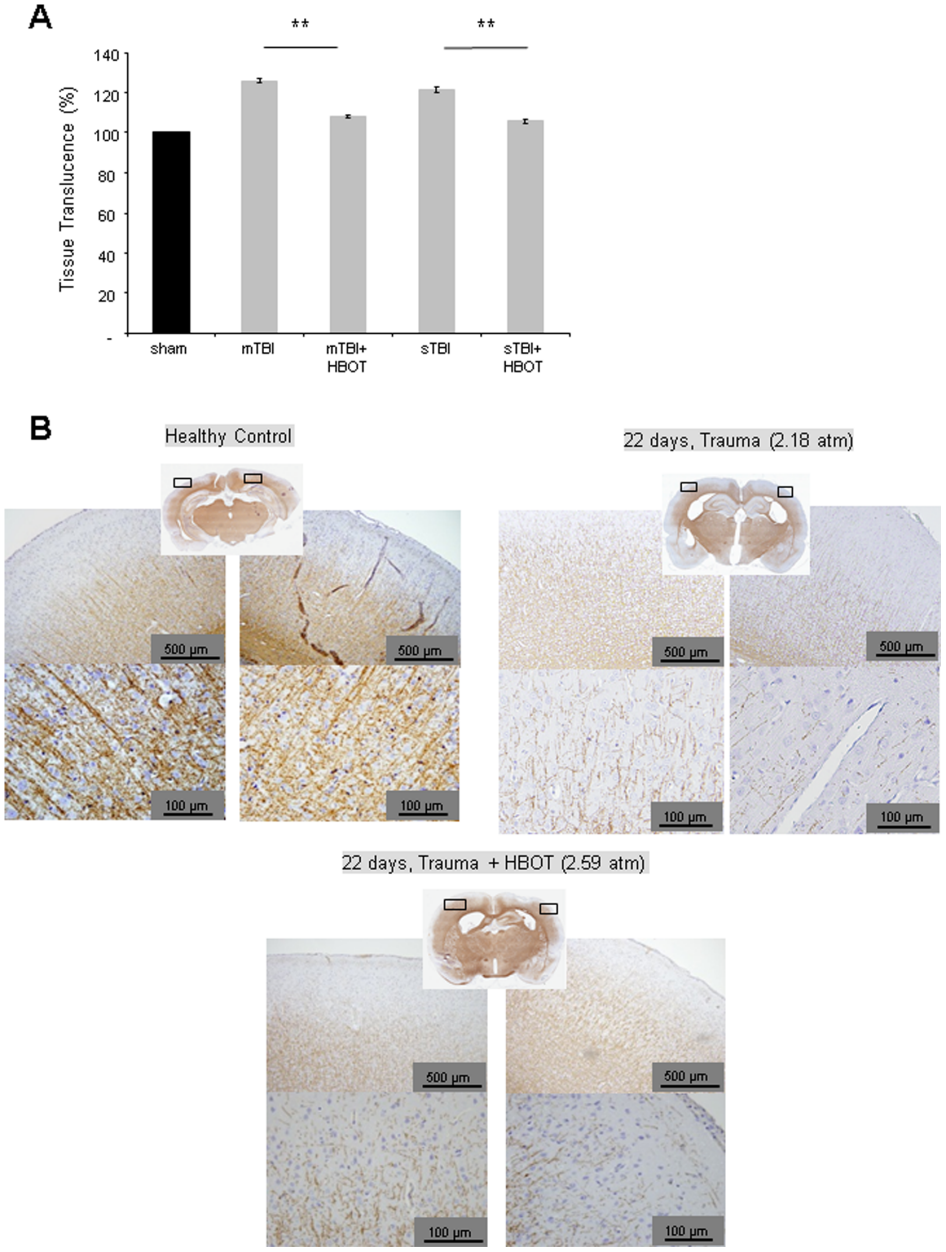
Modulation of myelin in the ipsilateral cortex at day 22 following induction of traumatic brain injury and HBO treatment. A. Quantitative analysis of myelin stained by Luxol Fast Blue. The ipsilateral hemisphere was analysed as a whole in order to avoid bias. Representation of the % tissue translucence of the ipsilateral hemisphere as compared to sham controls; a minimum of 4 successive brain slices per animal were analysed, *p<0.05, HBO-treated versus untreated animals; B. Exemplification of proteolipid protein (PLP) staining at day 22 following induction of traumatic brain injury and HBO treatment; number of animals see Table 1. Error bars represent ± standard error means.

The distinct HBOT-associated increase in MBP isoform expression 22 days post-injury was accompanied by a remyelination in the ipsilateral cortex. Statistically significant augmentation of myelin sheet in the ipsilateral cortex following three weeks of HBO treatment of the severely injured group was demonstrated by an increase in Luxol Fast Blue staining (Fig. 5A). Furthermore, proteolipid protein (PLP), a predominant myelin protein in the central nervous system, that was down-regulated in the ipsilateral cortex following trauma, was detectable again after 22 days of HBO treatment by immunohistochemistry (Fig. 5B).

### Effect of long-term HBO treatment on sensorimotor functions

Lateral fluid percussion injury resulted in pronounced cerebral damage of the ipsilateral sensorimotor cortex that was associated with a significant decrease in the amplitude latency as assessed by SEP. We therefore evaluated the motor abilities of the animals following injury and repetitive long-term HBO treatment. Sensorimotor function was evaluated by the Rotarod test that demands complex movement and coordination abilities and the composite neuroscore that consisted of forelimb reflex, hindlimb flexion, lateral pulsion, and angle board tests [54].

**Figure 6:**
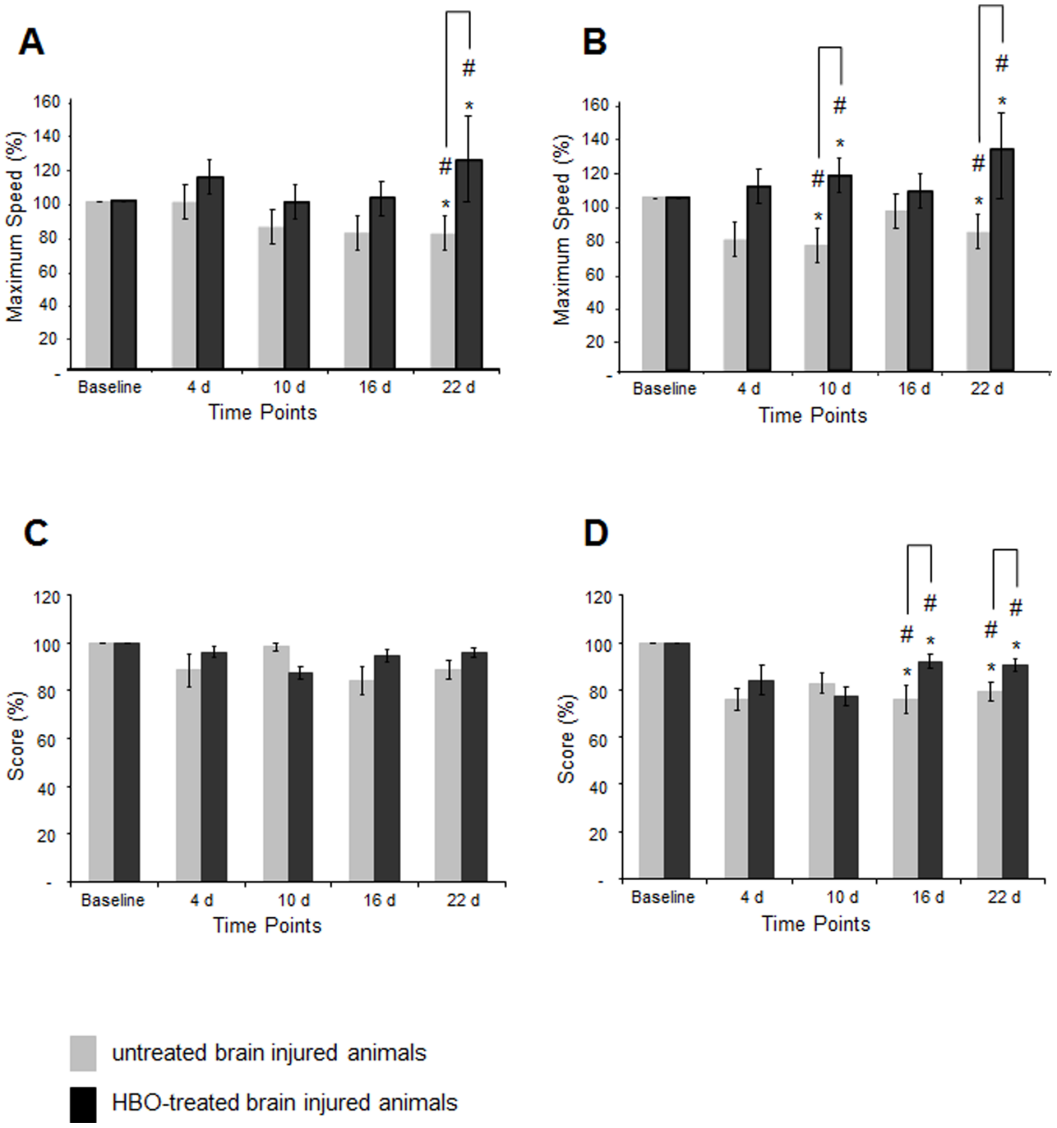
Time-dependent modulations of sensorimotor function following traumatic brain injury and HBO treatment. A: percentage of maximal speed output on the Rotarod of HBO-treated and untreated animals following moderate brain injury as compared to baseline performance; B: percentage of maximal speed output on the Rotarod of HBO-treated and untreated animals following severe brain injury as compared to baseline performance; C: percentage modulation in the composite neuroscores of HBO-treated and untreated animals following moderate brain injury as compared to baseline performance; D: percentage modulation in the composite neuroscores of HBO-treated and untreated animals following severe brain injury as compared to baseline performance; squares: untreated brain injured animals; circles: HBOT-treated brain injured animals; *p<0.02 (repeated measures mixed model ANOVA, *F(1,24) = 7.00, p<0.02*); ^#^ *p*<0.05 (t-test); Rotarod: n≥7 for each of the moderately injured groups, untreated and HBO-treated; n = 13 for each severely injured group, untreated and HBOT-treated; Composite Neuroscore: n≥11 for each group, moderately or severely injured animals with or without HBO treatment. (Note that uneven group sizes arise from missing data points in some animals, which were excluded in repeated measures mixed model ANOVA). Error bars represent ± standard error means.

Due to individual differences in baseline performance (results obtained for each animal before traumatic brain injury) post-injury performance on the Rotarod was analysed as percentage of the baseline values. An injury–induced, time-dependent decrease in the maximal speed maintained by the animals was observed in moderately and severely injured rats (Fig. 6A, B).

It should be taken into account that Rotarod performance is training dependent. Accordingly, the injury-induced sensorimotor impairment was statistically significant at each time point in severely injured animals as compared to the performance of sham animals at the different time points (Fig. S3).

Sensorimotor function impairment was compensated by HBO treatment. Among severely injured animals, the HBO-treated group showed a significantly better performance overall (Fig. 6B; *F(1,24) = 7.00, p<0.05*). The most pronounced HBOT-mediated compensation was observed after three weeks of repetitive treatment independent of injury severity (Fig. 6A, B). At this time point the performance of treated animals was comparable to the performance of the trained uninjured sham controls (Fig. S3).

Composite neuroscores were not significantly affected following induction of moderate trauma. Accordingly the effect of HBO treatment in this group was inconsequential (Fig. 6C). However, the significant reduction in composite neuroscores observed in severely brain-injured animals was compensated by the long-term HBO treatment at week two and three (*p*<0.03 and *p*<0.05, respectively) (Fig. 6D).

## Discussion

Hyperbaric oxygen treatment in a clinical setting is usually implemented in the form of repetitive sessions over extensive time periods in order to improve neurological outcomes following traumatic brain injury [55].

The results presented here demonstrate for the first time some of the potential mechanisms underlying the protective effect of repetitive long-term HBO treatment in an experimental traumatic brain injury model. Our analysis was focused on cerebral tissue modulations and associated functional improvements that are potentially related to the significant improvement of somatosensory-evoked potentials observed after three weeks of treatment.

SSEPs have been widely used at intensive care units since the late 1970s to supplement clinical, radiological and other parameters in assessing neurological function and outcome of patients suffering severe traumatic brain injuries [56]–[58]. The central conduction time (CCT) proved to be an important parameter of SSEP in assessing the neurological function of the brain following traumatic brain injury, because it strongly correlates with the impact of the initial trauma and proved to predict neurological outcome [56], [59].

A delay in CCT observed during trauma or in demyelinating diseases has been linked to conduction changes of the subcortical fiber tracts [60]. Vice versa, CCT decrease can be measured as a result of the remyelination of subcortical fiber tracts [57], [60]–[67]. Modulation of CCT following cerebral injury or therapeutic interventions might thereby be closely linked to demyelination or remyelination processes, respectively.

Extensive traumatic brain injury-associated white matter demyelination has been reported using animal models [68], [69] and in human studies [70].

In our experimental setting, a significant decrease in CCT was observed in the ipsilateral cortex of brain injured rats at late stages of disease progression after three weeks of repetitive HBO treatment. The significance of treatment duration for improvement of neurophysiological function is underlined by a study that reported the protective effects of short-term HBO treatment on cerebral integrity but no recovery of evoked potentials in brain injured rabbits following short-term treatment [71]. In the contralateral cortex exhibiting less pronounced cerebral damage, a spontaneous improvement of CCT was observed within this time frame that was not affected by HBOT. The spontaneous improvement of the CCT in the contralateral hemisphere is in accordance with observations that report inflammation-induced default remyelination in the central nervous system [72]–[74].

The time-dependent modulation in myelin basic protein expression observed in the ipsilateral cortex by western blot analysis indicates the HBOT-mediated improvement of CCT to be potentially due to remyelination processes. Significant injury and/or HBO treatment changes in expression patterns were mainly observed for the isoform 18.5-kDa and the exon II containing isoform 21.5-kDa. The 18.5-kDa isoform predominates in the adult CNS and is found in cell processes of mature oligodendrocytes (OLGs) that have migrated into axonal pathways where it is redistributed into the myelin sheaths [75]–[77]. The exon II containing 21.5-kDa isoform has been shown to be expressed at early stages during OLG differentiation where it is targeted to the nucleus and has been implicated in early differentiation processes such as proliferation and neurite outgrowth [75], [78], [79]. The isoform is also expressed in highly differentiated oligodendrocytes. It has been associated with protective processes such as inhibition of apoptosis and myelin turnover [75].

The impact of HBO seemed to be biphasic with rapid but less pronounced changes in MBP isoform expression after 4 days and significant modulations after two and three weeks of treatment. It should be noted that despite a progressive delay of CCT in week 1 and 2 following trauma, a noteworthy decrease in the expression of the MBP isoforms was not detected at these time points. This is in accordance with studies that have only observed a down-regulation of the phosphorylated isoform 21.5-kDa and 18.5-kDa in a spontaneously demyelinating mouse model [80]. Furthermore in early multiple sclerosis studies up-regulation of isoform 21.5-kDa was reported [81].

Up-regulation of MBP isoform expression was accompanied by an increase in Luxol Fast Blue staining in the ipsilateral cortex. These observations underline the notion of an HBO-associated recovery of fibre tracts by remyelination as indicated by the decrease of CCTs following three weeks of treatment.

HBO treatment-associated neurophysiological recovery was accompanied by a pronounced and statistically significant improvement in neuronal function of brain injured rats.

A pronounced and statistically significant recovery in neuronal function has been reported in a variety of studies implementing HBO treatment following brain injury. In most of these studies neurological function was shown to significantly improve within hours of early treatment often after one treatment session [82]–[89]. Although there was a distinct tendency of behavioural advancement at 4 days following three consecutive HBOT sessions in our study, a statistically significant improvement in sensory motor function of severely injured animals as compared to untreated animals was observed at the earliest after 10 days and six consecutive HBO sessions. However it should be noted that the maximum speed the injured HBO treated animals were able to control on the Rotarod was comparable or beyond the speed maintained before injury, indicating an enhancing effect of HBO treatment on the training abilities of the animals. An improvement of neurological function exceeding the abilities of the animals before injury has not been shown in any of the other studies analysing the protective effect of HBO treatment following brain injury. It has to be taken into account though that a direct comparison of the results is problematic, since injury models, HBO treatment modalities and behavioural and comparative group analysis varies considerably in different studies. In ischemic injury models, neurological function is often evaluated by score systems [83], [84], [86]–[88]. A statistically significant improvement of behavioural performance detected by composite neuroscore was also demonstrated after long-term (16 and 22 days) and repetitive (9 to 12) HBO treatments. We hypothesize that the lack of statistically significant augmentation of neuronal function during early HBO treatment in our study might be due to the high inter- and intragroup variation in the behavioural capacity. Repetitive and long-term HBO treatment seemed to be required to induce robust recovery.

Experimental studies analysing the effect of HBO treatment on cerebral function can support the understanding of mechanisms that might underlie the protective effects observed following HBO treatment of trauma patients. Our results indicate that HBO treatment might augment neuronal and neurophysiological function in damaged cerebral tissue due to remyelination events. Our results also indicate that these regenerative processes are based on the repetitive long-term HBO treatment of the injured animals. However, a direct extrapolation of these promising observations to trauma patients or patients suffering from demyelination diseases should be regarded with caution. In order to translate these experimental observations into clinical settings it is a prerequisite to understand the particular cerebral conditions that allow for the HBO-mediated induction of regenerative processes. In this standardized setting the first HBO treatment was administered immediately following trauma during the acute phase of the cerebral response. The perceptibility of the cerebral environment to HBO treatment during later stages of injury-induced inflammatory responses or during chronic cerebral inflammation has yet to be shown.

## Materials and Methods

### Ethics Statement

This study was carried out in strict accordance with the recommendations in the Guide for the Care and Use of Laboratory Animals of the Medical University of Graz. The protocol was approved by the Ministry for Science and Research, Department for Genetic Engineering and Animal Experiments (Permit Number: BMWF-66.010/0051-II/3b/2013). All surgeries were performed under sodium pentobarbital anaesthesia, and all efforts were made to minimize suffering.

### Study Design

Male Sprague-Dawley rats weighting 320–330 g were given access to food and water ad libitum. All animal procedures conformed to the guidelines of local and state animal protection guidelines. All efforts were made to minimize animal discomfort. The animals were allocated into following groups: (1) sham-operated animals, (2) moderate TBI, (3) moderate TBI with HBOT, (4) severe TBI and (5) severe TBI with HBOT. The grouping of animals into moderate or severe TBI was based on the pressure intensity implemented during lateral fluid percussion. Pressure intensities lower than 2.5 (2.1–2.4 atm.) atmospheres were defined as moderate; pressure intensities equal to or higher than 2.5 (2.5–2.8 atm.) atmospheres were defined as severe trauma as previously described [45]–[49]. Brain injured animals were analysed in the respective groups (severe, moderate) when trauma severity correlated with the analysed modulations. If there was no correlation, groups were combined (see data analysis in results). The different groups, number of animals per group and testing parameters are summarized in Table 1. Repetitive long-term HBO treatment was implemented as follows: animals were treated at day 1 (1 hour post-trauma), 2, 3, 7, 8, 9, 13, 14, 15, 19, 20 and 21, following trauma induction. Neurological tests, SEP-measurements and MRI scans were performed in the days following treatment (4, 10, 16 and 22). Brain tissue for IHC and western blots were harvested at the same respective time points (see Fig. 1).

**Table 1:** Grouping of animals and number of animals per group and testing parameter.

Groups	MRI^#^	SSEP^#^	Sensorimotor function^#^	IHC/ LFB	Protein Expression (WB)			
Time				*day*	*day*	*day*	*day*	*day*
	*continuous*	*continuous*	*continuous*	*22*	*4*	*10*	*16*	*22*
TBI	6*	12	*mTBI,* 9* *sTBI,* 12	6	5	5	5	5
TBI+ HBOT	10*	12	mTBI, 9* sTBI, 12	6	5	5	5	5
Sham	-	-	7	6	5	5	5	5

*Uneven number of animals is due to trauma- or anaesthesia-related loss of rats. ^#^Different groups were implemented since this allowed for simultaneous analysis of these parameters. Biases in the results of the distinct testing parameters due to previous handling of the animals were thereby avoided, i.e. anaesthetics and interventions during MRI or SSEP did not affect behavioural potentials of the rats and vice versa. mTBI: moderate traumatic brain injury; sTBI: severe traumatic brain injury.

All analyses in the study were performed blinded. Grouping of animals, induction of trauma and outcome analysis were performed by different researchers. For outcome analysis, the number of animals but no information about grouping or treatments was disclosed.

### Lateral Fluid Percussion Injury and HBO Treatment

Animals were anaesthetized with Isofluorane (1-Chlorine-2,2,2-trifluorineethyl difluorinemethylether) in an induction chamber (Rothacher) with flow rate of 2.5Vol% and 2.5 l/min consisting of Isoflurane and oxygen. To keep up anaesthesia, FDD-solution (2 ml Fentanyl, 2 ml Dormicum and 1 ml Dormitor; 0.1 ml/100 g body weight) was injected intraperitoneally. Animals were fixed in a stereotactic frame and an axiolateral craniotomy (4 mm in diameter) was performed between lambda and bregma over the right parietal cortex. A pre-primed Luer-Lock cap was fixed with dental cement. In sham animals, surgery was finished after craniotomy. For Lateral Fluid Percussion (LFP)-induced injury, animals were connected to the LFP-device (Am Scien Instruments, USA) via the Luer-Lock cap. A solution of sodium chloride was quickly infused into the closed cranial cavity, whereby the intracranial pressure increased (within a time-span of 21–23 ms) resulting in a temporary dislocation and deformation of underlying brain tissue. Moderate TBI was defined as trauma strength of 2.1–2.5 atmospheres (atm), severe TBI as 2.5–2.9 atm. Animals were treated with antibiotics (Cefotaxim, 0.05 g/ml/kg body weight, s.c.) on the first three days to prevent infection and with analgesics (Rimadyl, 10 mg/kg body weight, s.c.) for two days following injury.

One hour after induction of TBI, HBO treatment was implemented at 2.2 atmospheres (atm) and 100% oxygen for 1 hour and repeated as described above.

### Brain tissue harvesting and perfusion

According to the study schedule (for respective time-points of investigation see Fig. 1), the animals were narcotized and sacrificed by intraperitoneal injection of thiopental (Sandoz, 250 mg/ml). The calvaria were opened and ipsilateral and contralateral cortex were harvested. Tissue samples were put into Eppendorf tubes, shock-frozen in liquid nitrogen and stored at −80°C for further investigations (western blot). For immunohistochemical analysis of cerebral tissue, the thorax was opened and the still beating heart was exposed. The animal was transcardially perfused (via left chamber) with a perfusion solution (4% paraformaldehyde +1% picric acid). After overnight fixation at +4°C, the whole brain was harvested and embedded into paraffin-blocks for further investigations.

### Magnetic Resonance Imaging (MRI)

Magnetic Resonance Imaging (MRI) was used to assess the course of macro-structural tissue changes in the injured brain with/without HBOT. The animals were examined on a 3.0T whole body system (Tim Trio, Siemens Medical Systems, Erlangen) which was retrofitted for animal MRI. The MR protocol consisted of a three-dimensional T2-weighted fast spinecho (FSE)-sequence (in-plane resolution 200×200 µm^2^) and a spoiled 3D T1-weighted gradient echo (FLASH) sequence (in-plane resolution 200×200 µm^2^) for structural imaging. The acquisition time was approximately 30 minutes during which time the animals were kept under anaesthesia with Isofluorane.

### Somatosensory Evoked Potentials (SSEPs)

The animals were anaesthetized with FDD-solution (2 ml Fentanyl, 2 ml Dormicum and 1 ml Dormitor; 0.1 ml/100 g body weight). After induction of general anaesthesia, animals were transferred to a heating mattress to maintain normal body temperature and subdermal needle electrodes were placed subcutaneously on the head. The recording sites were defined points adapted for use in rats according to the human 10–20 EEG system [50] as followed: C3': half the distance between the midline and the left external acoustic meatus in a line between the left and right external acoustic meatus. C4': half the distance between the midline and the right external acoustic meatus in a line between the left and right external acoustic meatus. Fpz: half the distance between the nasion and the bregma in the midline. Cerv: in the midline over the dorsal neck. A ground electrode was inserted subcutaneously above the right abdomen.

Two stimulating electrodes were put subcutaneously in the proximal part of the tail one centimetre apart from one another, whereas the distal electrode served as the anode and proximal as the cathode. Electrode impedances were measured and controlled for values below 5kOhms. Biphasic square wave pulses of 0.5 milliseconds in duration and 9.0 milliamperes strengths, which meant supramaximal stimulation in all cases, were applied to the rats' tails with a frequency of 2.1 Hertz in all animals.

Somatosensory evoked potentials (SSEP) were recorded over three channels simultaneously, averaged, displayed and stored for later analysis, accordingly to the parameters shown below:

Display sensitivity: 2 µVolts/division; Band pass filter. 30–1500 Hertz; Channel 1: C3'-Fpz; Channel 2: C4'-Fpz; Channel 3: Cerv-Fpz; Averaging: 100 times.

### Western Blot

Cortical brain tissue samples were harvested, and protein and RNA extraction were done using mirVana PARIS Kit (Ambion) according to the manufacturer's instructions. Protein content was determined by the BCA protein assay kit (Novagen) using bovine serum albumin (BSA) as a standard. 20 µg protein lysate was mixed with 4x Sample Loading Buffer (Biorad) and 20x Reducing Agent (Biorad) heated for 5 minutes at 95°C and separated by SDS-PAGE in 12% Criterion XT Bis-Tris precast gels (Biorad). After transfer (1 hr, 40V) to a nitrocellulose membrane (Whatman), membranes were blocked (StartingBlock T20 blocking buffer, Thermo Scientific) for 15 minutes at room temperature and incubated overnight at 4°C with 0.2 µg/ml polyclonal rabbit anti-myelin-basic-protein antibody (Genscript). Membranes were rinsed again with 0.1% TBST then incubated with 0.2 µg/ml horseradish peroxidase-conjugated goat anti-rabbit IgG antibody (Santa Cruz Biotech) for 1 hr at room temperature. Signals were detected using SuperSignal West Pico Chemiluminescent Substrate (Thermo Scientific) and quantified using Bio-Rad2000 gel imaging system with QUANTITY ONE software (Biorad). Monoclonal mouse anti-beta actin antibody conjugated to HRP was used as a loading control at a concentration of 31 ng/ml.

### Immunohistochemistry

Formalin-fixed paraffin-embedded tissue (FFPE) sections were deparaffinized in an incubator at 65°C for 40–60 minutes followed by incubation in xylene (5 min +10 min, twice) and xylene-EtOH mixture (1∶1) for another 5 min. Sections were rehydrated by incubation in decreasing alcohol concentrations. Microwave treatment at 90W for 20 min in 10% target retrieval solution pH 6 (Dako Österreich GmbH) was followed by endogenous peroxidase blocking through incubation in 3% H_2_O_2_ for 10 min. To reduce unspecific antibody binding, blocking with serum-free protein block (Dako Österreich GmbH) was performed for 30 min. Sections were incubated with anti-proteolipid protein (PLP) antibody (1 µg/ml) (Chemicon (Millipore)) and isotype control (1 µg/ml) overnight (+4°C). Sections were subsequently incubated with a secondary antibody anti-IgY (host rabbit) (Abcam) for 1 hour and reactivity was visualized with UltraVision LP large-volume detection system HRP polymer (Thermo Fischer Scientific, Freemont, CA, www.labvision.com) and stained with Dako REAL DAB+ Chromogen (Dako Österreich GmbH) for 1 min following the manufacturer's instructions. Stained sections were counterstained with Harriś Hematoxylin solution (Merck) for 20 seconds, blued in hot tap water and mounted with mounting medium (Tissue Tek, Coverslipping Resin, Sakura Finetek USA Inc, Torrance, CA; www.sakuraus.com).

### Luxol Fast Blue Staining

Formalin-fixed paraffin-embedded tissue (FFPE) sections were deparaffinized in an incubator at 65°C for 40–60 minutes and rehydrated to 95% ethyl alcohol. The slides were incubated in Luxol Fast Blue solution (0.1% in 95% ethyl alcohol) at 56°C overnight. Subsequently the slides were cooled down to room temperature and washed in 95% ethyl alcohol and distilled water. Subsequently samples were incubated in a lithium carbonate solution (0.1%) for 5 minutes in 70% ethyl alcohol until the grey matter was clear and the white matter was sharply defined. The slides were placed in distilled water. After dehydration the slides were mounted with mounting medium (Tissue Tek, Coverslipping Resin, Sakura Finetek USA Inc, Torrance, CA; www.sakuraus.com) and pictures were taken with Aperio (Aperio ePathology). Stainings from a minimum of 4 successive slices per brain were quantitatively analysed. Quantitative analysis of ipsilateral hemisphere translucence was performed using Adobe Photoshop CS5 extended: images at the same magnification were straightened and the size of each image was adjusted to 720×540 pixels. The area of the entire ipsilateral hemisphere was selected by a rectangular marquee. The size of the marquee was saved and used for all images. Translucency of the selected areas was determined in the RGB setting at 0.299 - red, 0.587 - green, 0.114 - blue.

### Neurological Sensorimotor and Cognitive/Behavioural Tests

Animals were tested three days before the trauma in order to habituate them to the procedure and to be able to record their optimal performance (baseline performance). Subsequently behavioural tests were performed at days 4, 10, 16 and 22 post-injury.

### Rotarod

Animals were put on a computer-controlled roller with defined starting-, ending- and maximum speeds. Speed was recorded in rounds per minute [51]. The experiment was stopped after 5 minutes or when the animal fell off of the roller. Thereby, the readout of the experiment was the maximum speed an animal could tolerate on the Rotarod without falling off. The experiment was repeated four times at each time point. The best test result was used for the statistical analysis. It also reflected the potential maximum performance level of each animal (proof of principle) and avoided bias by including data that was, for example, due to an animal's active jumping from rather than falling off of the Rotarod. Maximum performance was achieved randomly at different times of the repetitive trial and was thereby not due to learning effects or fatigue.

### Composite Neuroscore

For the composite neuroscore, a combination of different testing methods [51], [52] was analysed: (1) function of right and left forelimb when pulling the animal up by its tail, (2) function of right and left hindlimb when pulling the animal up by its tail, (3) resistance of the animal when pushing it laterally to the right- and left-hand side, (4) ability of the animal to stand on an angled plate; the measurement is of the highest possible angle where it is still able to hold on. For each function the animals could reach a maximum score of 4 points, which made a total of 16 points. Animals were only tested once at each time point.

### Statistical Analysis

We used repeated measures mixed model ANOVA for continuous variables (SEP, Rotarod testing) to assess within subject differences (time) and between subject differences (HBO treated or not treated). For analysis of composite neuroscore, sample groups did not meet some important testing assumptions for mixed model ANOVA (e.g. sphericity). Furthermore, detected interaction also hampered accurate interpretation of the test results. Therefore, we followed another strategy: Scores at different time points were compared by repeated measures ANOVA to assess within subjects differences after TBI. Then the question of whether HBOT makes any change at given time points was assessed by single t-tests. In the latter, we minimized the test numbers to minimize Type I error. Western blots and for luminescence measures of Luxol Fast Blue, sections were assessed by t-test. For SEP analysis, actual values were used; for behavioural test analysis, percentages were calculated. Results before traumatic brain injury were taken as 100%. The reason for this is that different animals showed different baseline performances and to be able to compare their performance increase or decrease, working with percentages was essential. Myelin basic protein expression values and luminescence measures of Luxol Fast Blue sections were also analysed as percentages of sham controls.

A p-value ≤0.05 was considered significant and ≤0.001 was considered highly significant.

Correlation coefficients were calculated to describe the relationship between two different parameters (e.g. trauma strength and outcome in behavioural tests). An r-value of >0.55 or <−0.55 was considered a meaningful correlation.

## Supporting Information

Figure S1 Correlation coefficiency of SSEP and trauma severity at the different time points of measurement. Light grey bar: brain injured animals; dark grey bars: HBO-treated brain injured animals.
(PDF)

Figure S2 Correlation coefficiency of sensorimotor function and trauma severity at the different time points of measurement. A correlation coefficiency (r>-0.5) was observed for sensorimotor abilities (Rotarod and Compsite neuroscores) for brain injured animals. The results are therefore analysed in groups of moderately and severely injured rats; light grey bar: brain injured animals; dark grey bars: HBO-treated brain injured animals.
(PDF)

Figure S3 Time dependent modulations of the maximal speed output on the Rotarod of brain injured (grey bars), HBO-treated brain injured animals (white bars) and sham controls (Black bars) as compared to the baseline performance of each animal. * *p*≤0.05.
(PDF)

Figure S4 Correlation coefficiency of MBP isoform expression and trauma severity at the different time points of analysis. A. 21.5-kDa isoform; B. 18.5-kDa isoform; C. 17.2-kDa isoform; A correlation coefficiency of trauma severity and MBP isoform expression was only observed at distinct time points for some isoforms. For quantitative analysis results obtained from severely and moderately injured animals were combined.
(PDF)

Table S1 Number of animals with distinct lesions (epiduralhaematoma, atrophy of the left ventricle, atrophy of the right ventricle, cortical destruction) in HBOT- or control group at 4 days or 3 weeks post-injury.
(PDF)
