# Supplementary figures and images for: Repetitive Long-Term Hyperbaric Oxygen Treatment (HBOT) Administered after Experimental Traumatic Brain Injury in Rats Induces Significant Remyelination and a Recovery of Sensorimotor Function
Source: PLoS One. 2014 May 21;9(5):e97750. doi: 10.1371/journal.pone.0097750 (PMC4029808; doi:10.1371/journal.pone.0097750)

**A**

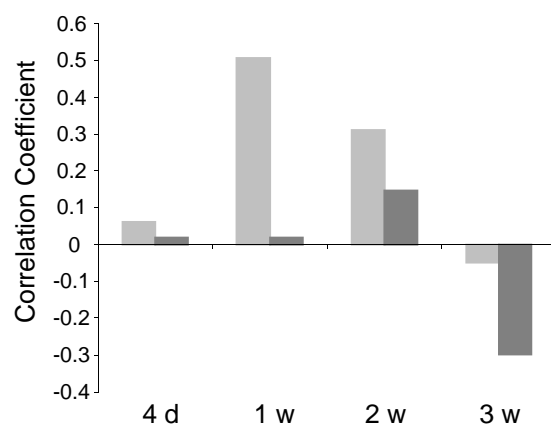

Figure S1

Supplement: Figure S1 — Correlation coefficiency of SSEP and trauma severity at the different time points of measurement. Light grey bar: brain injured animals; dark grey bars: HBO-treated brain injured animals. (PDF) [file pone.0097750.s001.pdf]

**A**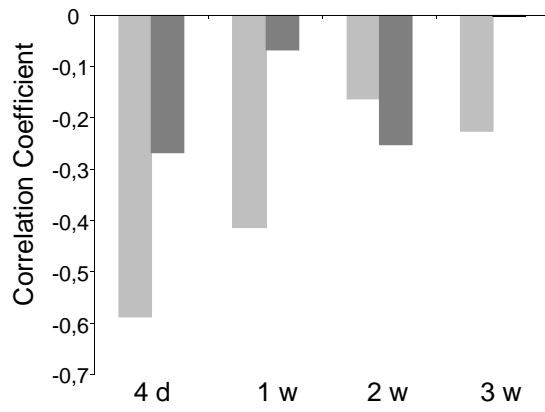**B**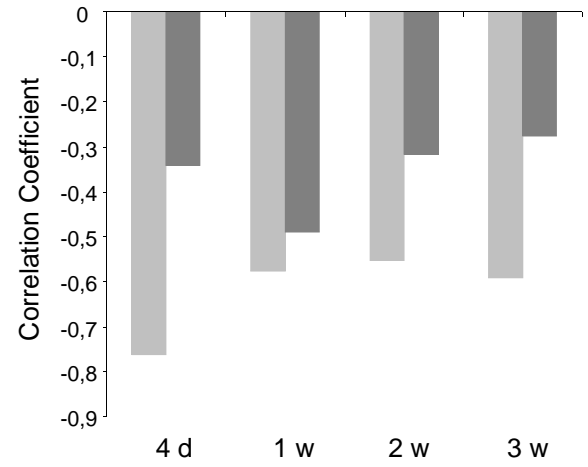

Figure S2

Supplement: Figure S2 — Correlation coefficiency of sensorimotor function and trauma severity at the different time points of measurement. A correlation coefficiency (r>-0.5) was observed for sensorimotor abilities (Rotarod and Compsite neuroscores) for brain injured animals. The results are therefore analysed in groups of moderately and severely injured rats; light grey bar: brain injured animals; dark grey bars: HBO-treated brain injured animals. (PDF) [file pone.0097750.s002.pdf]

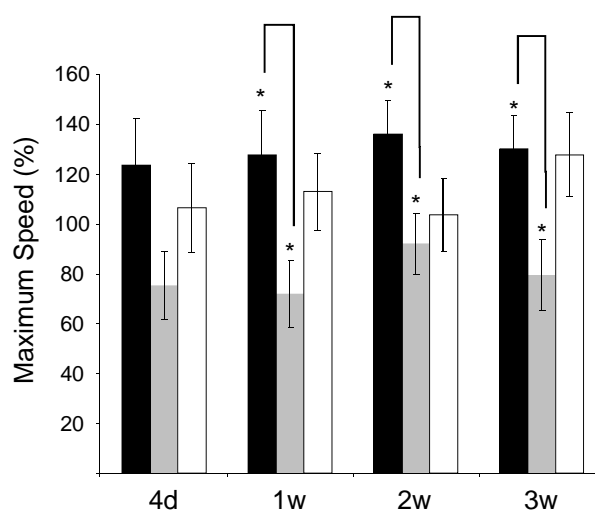

Figure S3

Supplement: Figure S3 — Time dependent modulations of the maximal speed output on the Rotarod of brain injured (grey bars), HBO-treated brain injured animals (white bars) and sham controls (Black bars) as compared to the baseline performance of each animal. * p≤0.05. (PDF) [file pone.0097750.s003.pdf]

**A**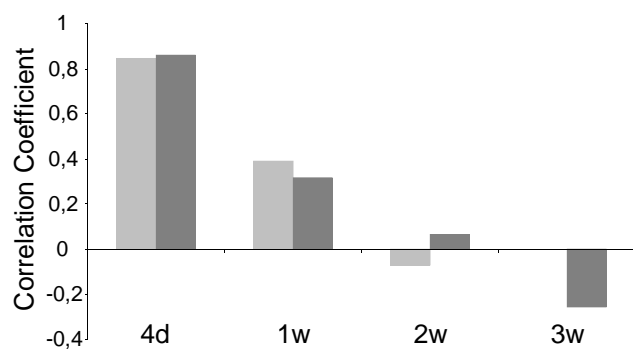**B**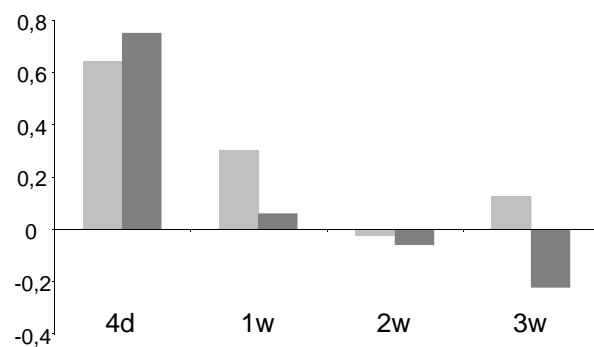**C**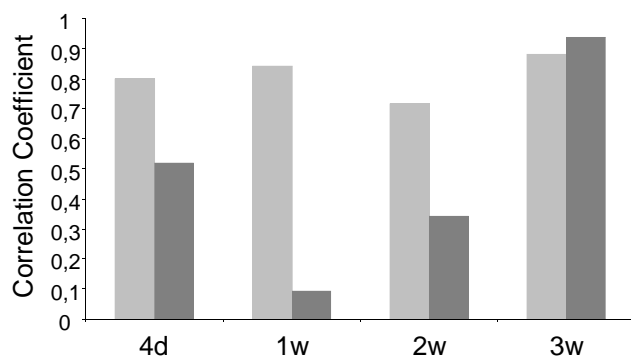

Figure S4

Supplement: Figure S4 — Correlation coefficiency of MBP isoform expression and trauma severity at the different time points of analysis. A. 21.5-kDa isoform; B. 18.5-kDa isoform; C. 17.2-kDa isoform; A correlation coefficiency of trauma severity and MBP isoform expression was only observed at distinct time points for some isoforms. For quantitative analysis results obtained from severely and moderately injured animals were combined. (PDF) [file pone.0097750.s004.pdf]
