# Supplementary material for: Repetitive Long-Term Hyperbaric Oxygen Treatment (HBOT) Administered after Experimental Traumatic Brain Injury in Rats Induces Significant Remyelination and a Recovery of Sensorimotor Function
Source: PLoS One. 2014 May 21;9(5):e97750. doi: 10.1371/journal.pone.0097750 (PMC4029808; doi:10.1371/journal.pone.0097750)
Supplement: Table S1 — Number of animals with distinct lesions (epiduralhaematoma, atrophy of the left ventricle, atrophy of the right ventricle, cortical destruction) in HBOT- or control group at 4 days or 3 weeks post-injury. (PDF) [file pone.0097750.s005.pdf]

	4 days		3 weeks	
	- HBOT	*+ HBOT*	*- HBOT*	*+ HBOT*
Epiduralhaematoma	1/6	2/10	0/6	1/10
Atrophy left ventricle	1/6	1/10	2/6	4/10
Atrophy right ventricle	1/6	2/10	4/6	5/10
Cortical destruction	1/6	4/10	5/6	6/10

Table S1
